# Self-Organizing Peer-To-Peer Middleware for Healthcare Monitoring in Real-Time

**DOI:** 10.3390/s17112650

**Published:** 2017-11-17

**Authors:** Hyun Ho Kim, Hyeong Gon Jo, Soon Ju Kang

**Affiliations:** 1 School of Electronics Engineering, College of IT Engineering, Kyungpook National University, 80 Daehakro, Bukgu, Daegu 702-701, Korea; whdxo10@naver.com; 2 Center of Self-Organizing Software-Platform, Kyungpook National University, 80 Daehakro, Bukgu, Daegu 702-701, Korea; tsana@ee.knu.ac.kr

**Keywords:** healthcare, real-time monitoring, self-organizing, P2P connection

## Abstract

As the number of elderly persons with chronic illnesses increases, a new public infrastructure for their care is becoming increasingly necessary. In particular, technologies that can monitoring bio-signals in real-time have been receiving significant attention. Currently, most healthcare monitoring services are implemented by wireless carrier through centralized servers. These services are vulnerable to data concentration because all data are sent to a remote server. To solve these problems, we propose self-organizing P2P middleware for healthcare monitoring that enables a real-time multi bio-signal streaming without any central server by connecting the caregiver and care recipient. To verify the performance of the proposed middleware, we evaluated the monitoring service matching time based on a monitoring request. We also confirmed that it is possible to provide an effective monitoring service by evaluating the connectivity between Peer-to-Peer and average jitter.

## 1. Introduction

As society rapidly ages globally, the health, safety and institutional issues of the elderly are attracting significant attention. In addition, as the number of chronic illnesses, such as cerebrovascular and cardiovascular diseases, increases with age, a new public infrastructure for elderly care is needed. Those suffering from a chronic disease require regular care and medical treatment. And, they need periodic monitoring by their guardians. In reality, however, problems such as rising medical and long-term care costs may arise.

To solve these problems, the Internet of Things (IoT) has been attracting the attention of the healthcare monitoring industry, which is developing services and solutions related to disease diagnosis, treatment and prevention. For example, a smart hospital system (SHS) [1] based on RFID and 6LoWPAN has not only tracked the location of patients and employees in the hospital but also offered a service that allows the remote caregiver to monitor the patient’s condition. Similarly, the Help to You (H2U) healthcare system [2] transmits multi-bio signals to a central data base via a smart phone and a doctor or guardian can access the central data base to monitor the health status of the patient. In addition, a pervasive patient health monitoring (PPHM) system [3] provides flexible and scalable remote health monitoring that integrates the capabilities of the IoT and cloud technologies for remote monitoring of a patient’s health status.

**Figure 1:**
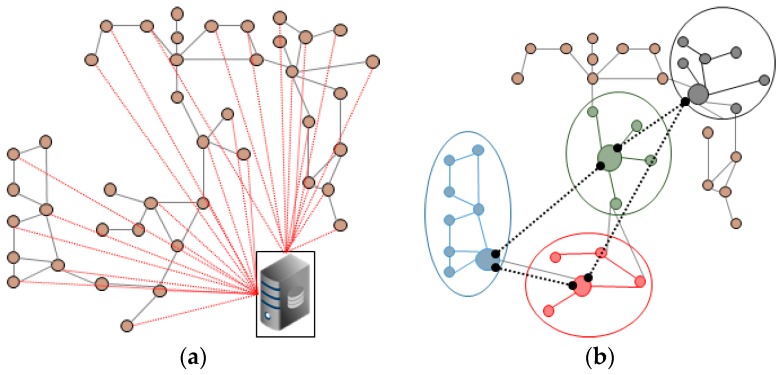
Traffic flow in a centralized platform and the self-organizing distributed platform. (**a**) Centralized Platform; (**b**) Self-Organizing Distributed Platform.

The above systems and most of the current systems are being implemented by wireless carrier through centralized servers. As shown in Figure 1a, system with this structure can cause problems such as network congestion due to traffic concentration, service-delay problems caused by multi-hop communication and a high communication cost because the device is always connected with a centralized server. In addition, security problems may arise because the various biometric data of persons under care at remote locations are concentrated at a central server.

To solve these problems, we have previously proposed a fully distributed self-organizing middleware platform [4,5] without a central server. As shown in Figure 1b, this study autonomously groups the mobile nodes based on their location and shows neighboring communication through the representative nodes of the group without a central server. Based on this platform, we propose a healthcare monitoring middleware for transmitting multi-bio data to a remote caregiver without a central server using a self-organizing localized IoT messaging (SLIM) hub [6].

If SLIM hub of our proposed middleware is on a public network, monitoring and streaming services are available to the caregivers without a central server. However, if SLIM hubs are in a different private network environment, communication between SLIM hubs is impossible and monitoring services with the remote guardian are impossible. Currently, most homes are located in a private network environment using NAT (Network Address Translation) device [7] due to lack of IP addresses. So, the proposed middleware needs to recognize its own network environment in order to enable service in any network environment and if it is a private network environment, it needs peer-to-peer communication between hubs.

The main contributions of this paper are:

- Healthcare monitoring and streaming middleware based on self-organizing middleware platform that can monitor care recipient regardless of where the caregivers are located without a central server.
- Supports peer-to-peer connections for self-organizing middleware platforms to provide healthcare monitoring and streaming services in a private network environment.

The remainder of this paper is structured as follows. In Section 2, we introduce the previous related work. Section 3 provides an overview of a streaming service and the concept. Section 4 describes the detailed design of the streaming service, Section 5 evaluates its performance and Section 6 discusses the proposed service. Finally, some concluding remarks and future works are provided in Section 7.

## 2. Related Research

### 2.1. Self-Organizing Middleware Platform and Self-Organizing Localized IoT Messaging Hub

**Figure 2:**
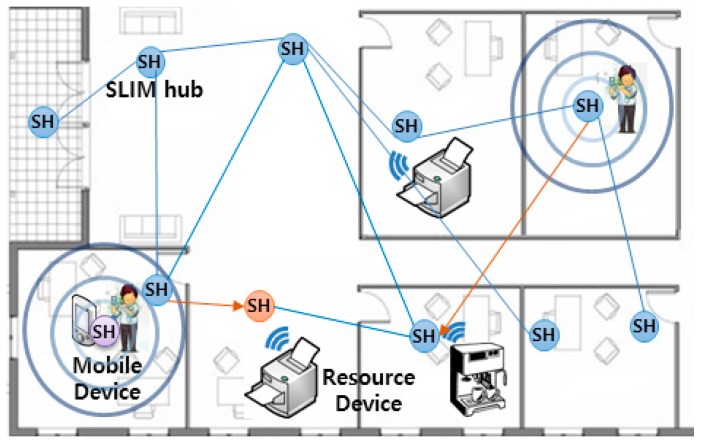
An example of a Self-Organizing Middleware Platform for a printing service.

A self-organizing middleware platform [4,5] is an indoor location-based service platform based on an overlay network and is autonomously serviced without direct manipulation, such as the initial settings required by the users. In addition, it is a platform that recognizes the surrounding environment and provides optimal indoor location-based services through autonomous collaboration between nodes without a central server. For example, the person on the left in Figure 2 uses a mobile device to find the nearest printer in his neighborhood. The user is provided with the service of the nearest printer through the cooperation of nodes (SLIM hub) which exists in each indoor space. Research related to this area has focused on real-time indoor location-based search and services [8]. In [8], the authors propose a service that finds the nearest path between nodes and guarantees real-time QoS. Another research [6] suggests a physical distance-based asynchronous messaging platform that specializes in processing personalized data and location-based messages.

### 2.2. NAT Traversal

NAT is designed for IPv4 address conservation and allows every computer to be given a unique Internet address without diminishing the available pool of public IP addresses. This is made possible because a NAT router maps individual port information for public IP addresses into information that can be assigned to multiple internal private addresses.

**Figure 3:**
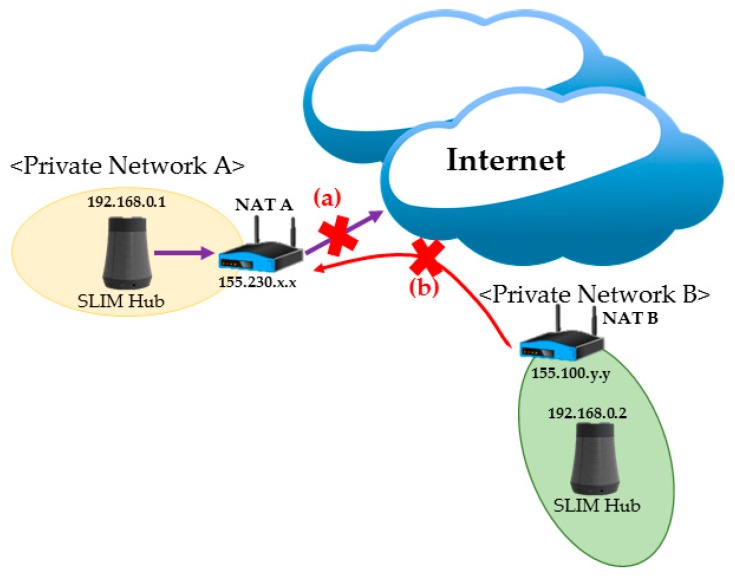
Problems of NAT device and private network.

However, there is a problem in communicating in another private network environment. For example, the SLIM hubs in Figure 3 reside under different NAT devices A and B and are in different private networks. Under this situation, when 192.168.0.1 of private network A tries to send a packet to 192.168.0.2 of private network B, the packet cannot be routed. In addition, if 192.168.0.2 of private network B sends a packet to a public IP (155.230.x.x) of NAT device A, the device will drop the packet. As a result, communication between logical neighbor nodes is impossible in a private network environment. That is, it is impossible to provide local-based or streaming services using a self-organizing middleware platform. To solve this problem, a number of technologies have been developed, including Universal Plug and Play, Application Level Gateway [9], Session Traversal Utilities for NAT (STUN, RFC 5389), Traversal Using Relay NAT (TURN, RFC 8155) and Hole Punching [10,11,12]. Through this study, we resolved this problem by using the ICE protocol (RFC 5245), which is a combination of STUN and TURN.

### 2.3. Healthcare Monitoring

Bio-signal streaming and healthcare monitoring system can be divided into sensor layer, networking layer, service and interface layer [13,14]. Similar to this approach, Wang [15] proposed a service consisting of three layers: sensor network, mobile network and remote monitoring network layers.

**Figure 4:**
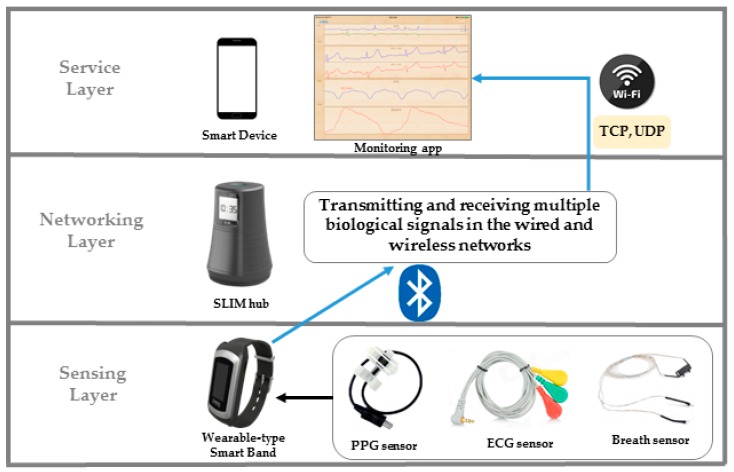
The service layer of the proposed system.

**Figure 5:**
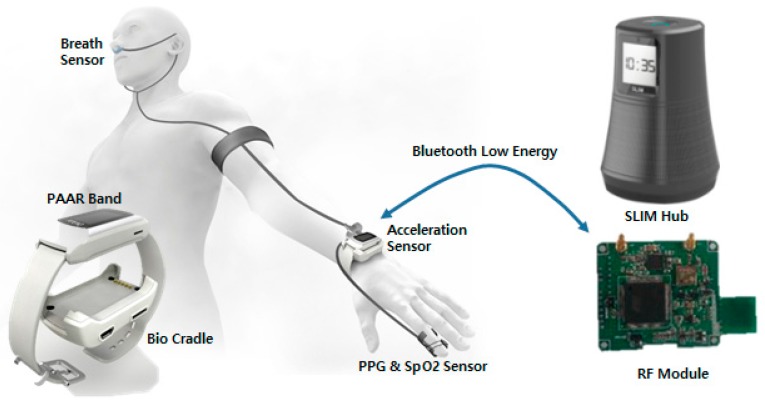
Multi-bio-signal measuring device and RF module of SLIM hub.

First, in a sensor network layer [16], the biometric data acquired by the bio-signal measurement device are transmitted to the mobile network layer using Zigbee Protocol. Unlike Wang, Khan, Laine [17,18] used Bluetooth as another way to build a sensor network. In addition, Passow [19] proposed a sensor network using ANT protocol. Second, the network layer supports sensor data collection and remote transmission of biometric data. In the network layer, gateway is required for sensor data collection and wireless transmission. There are studies [20,21,22] in which smart phones are acting as gateways or gateways are installed per unit space to collect bio-signals and transmit them to remote servers. Finally, the service and interface layers provide healthcare services [23] that enable users to easily understand health status by storing and analyzing biometric data in the cloud [24] and centralized servers [25]. However, with a centralized server architecture, if a single point of failure occurs, the entire system will stop when the server goes down. In addition, a security problem [26,27,28,29] may occur because biometric information is gathered in a single location. The real-time performance is degraded because locally occurring services go through a central server. In this paper, Figure 4 shows that the proposed middleware is divided into three layers according to the health care and IoT system distinction method. First, the sensor layer transmits biomedical signals measured by Personal Activity Assisting and Reminding (PAAR) band and Bio cradle [30] to a SLIM hub existing in each unit space using Bluetooth Low Energy (BLE). The second layer is networking layer, SLIM hubs in this layer stream to remote devices using TCP/IP (Transmission Control Protocol/Internet Protocol), UDP (User Datagram Protocol) and Wi-Fi. Finally, the service layer provides application programs such as biometric signal monitoring and interfaces that can be easily used by users. The left side of Figure 5 shows the configuration of the band and cradle and the bio-signal sensor connected. As shown on the right side of Figure 5, the biometric signal is transmitted to the module responsible for the RF communication of the SLIM hub using Bluetooth Low Energy (BLE) and finally transmitted to the destination without the central server.

## 3. Concept of Proposed Monitoring and Streaming Service

### 3.1. Overview of Proposed Monitoring and Streaming Service

**Figure 6:**
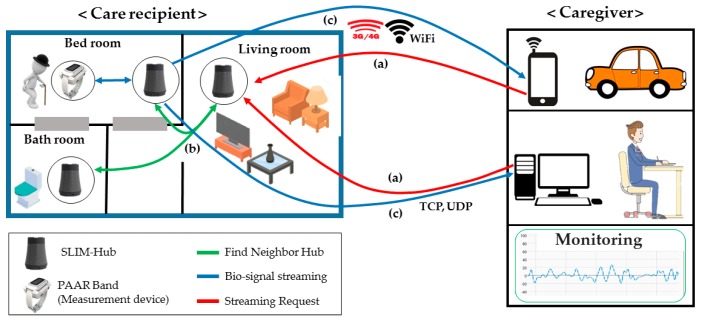
Monitoring and Streaming service scenario. (**a**) Monitoring and Streaming request; (**b**) Lookup neighbor SLIM hubs; (**c**) Streaming to the remote caregiver.

Figure 6 shows the basic scenario of the monitoring and streaming service proposed in this paper. It is assumed that a care recipient wears a PAAR band at home. SLIM hubs are located in each room (logical unit space). The right side of Figure 6 shows a caregiver moving in a car and at work. This indicates that the caregiver can verify the health status of the care recipient using a smartphone app under any circumstances. Suppose the caregiver’s monitoring app is registered with the representative SLIM hub of the care recipient. For example, in Figure 6, the SLIM Hub in the living room is the hub registered in the caregiver’s app and the SLIM hub in the other room is the unregistered hub. First, the caregiver sends a request to the SLIM hub located in the living room, which is registered in the app, to receive the health status of the care recipient, as shown in Figure 6a. If the care recipient is not located near the registered SLIM hub, the SLIM hub in the bedroom is searched using the unique ID of the PAAR band, as shown in Figure 6b. That is, if there is no band near the requested hub, the hub autonomously looks for a hub near the band. The requested PAAR band streams the biometric data to the SLIM hub. Finally, the SLIM hub streams to the remote guardian using TCP/IP or Wi-Fi/3G/4G, as shown in Figure 6c.

### 3.2. Concept of Streaming Service in a Public Network

**Figure 7:**
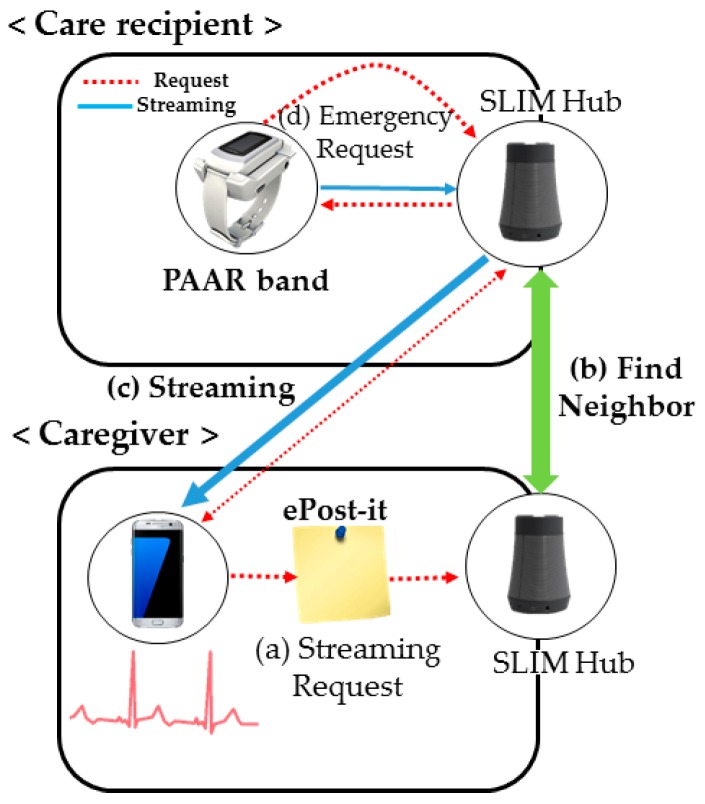
Sequence of streaming service operation.

**Figure 8:**
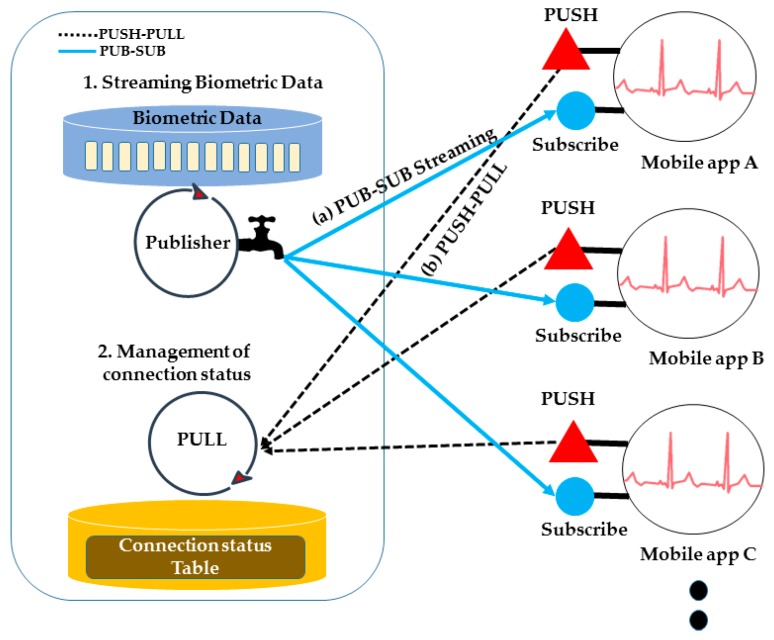
Bio-signal streaming and connection management.

In a public network environment, the service structure consists of three parts: request, streaming and connection management. First, even if the caregiver does not know the IP address of the SLIM hub where the care recipient is located, the ID of the PAAR band is transmitted in an ePost-it [6], as shown in Figure 7a (ePost-it is a location-based asynchronous messaging platform for implementing asynchronous messaging between various devices and services). Then, the SLIM hub that receives the request can find a SLIM hub within the vicinity of the PAAR band through neighbor collaboration, as shown in Figure 7b. The request is sent to the PAAR band worn by the care recipient and finally the streaming begins, as shown in Figure 7c. In addition, if the care recipient is in an emergency, the care recipient needs to notify the caregiver of the emergency message, as shown in Figure 7d. The SLIM hub receives the emergency message and sends a message to the registered caregiver. Second, the structure for sending biometric data to a remote caregiver uses the publisher-subscriber (PUB-SUB) model [31,32] of ZeroMQ [33]. In the PUB-SUB model, the publisher publishes the message without knowing what subscriber it is. The subscriber receives a message that fits the interest without any knowledge of the publisher. By separating the publisher and the subscriber, high scalability and dynamic network topology are possible. In addition, this model has an advantage in that the PUB can stream the acquired biometric data to a plurality of SUBs, as shown in Figure 8a. However, because the PUB does not know whether the SUB is normally connected, the PUSH-PULL model of ZeroMQ is used for management purposes. The SUB periodically sends the current status information and the received connection status management manager can manage a plurality of SUB states by storing and updating the corresponding information in a table.

### 3.3. Concept of Streaming Service in a Private Network

If the hub is on a private network and a device on the external network sends packets to the hub, it cannot be routed. In addition, according to the mapping and filtering rule [7] of the NAT device, incoming packets are dropped and communication between neighbors is impossible. To solve this problem, we use the ICE protocol, which is a combination of the STUN and TURN protocols. STUN protocol is used to find the public IP (Server Reflexive Candidates) and private IP (Local Candidates) mapped to the NAT device. If the result of STUN indicates that P2P communication is impossible, it should be relayed through a TURN server. Therefore, in case P2P communication does not work, it is assigned relay port from TURN server (Relayed Candidates).

**Figure 9:**
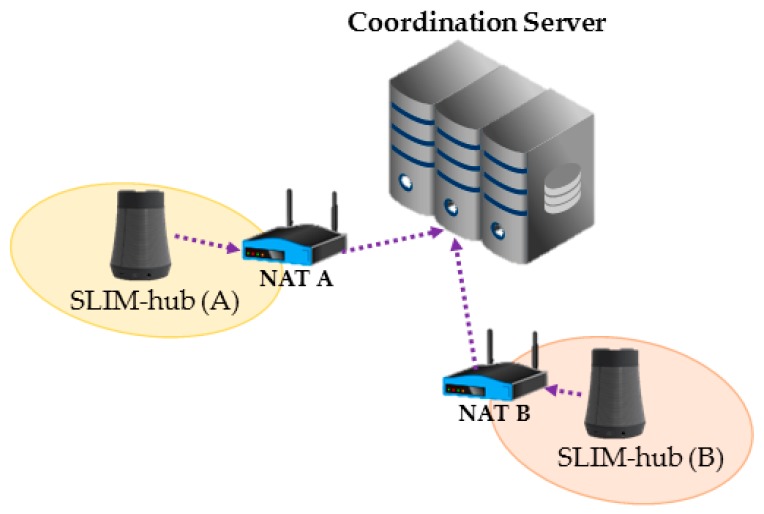
Register ID and local and public IPs, sent to the server.

This process is called “gather all candidates”. As shown in Figure 9, the SLIM hub that obtains the candidate address list transmits it to the coordination server consisted of a combination of STUN and TURN servers. The server then stores the state of each SLIM hub, as shown in Table 1.

**Figure 10:**
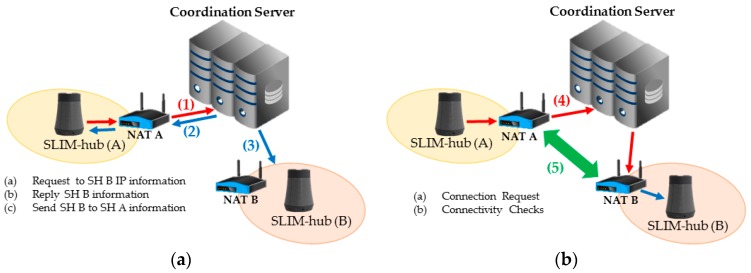
Connection process between the SLIM hubs in a private network. (**a**) Request IP information from another SLIM hub; (**b**) Connection request and connectivity checks.

As shown in Figure 10a, SLIM hub A requests the coordination server for IP information of SLIM hub B to send a streaming request to B (1). Then, the coordination server sends the IP information of B that was stored in a table (2) and simultaneously sends the IP information of A to SLIM hub B (3). Figure 10b shows the connection process. SLIM hub A sends a connection request message to the coordination server (4). The server that receives the request then sends a connection request message to B. Finally, A and B send a connectivity check message to all listed candidates of the other party (5). As a result, the connection between the two SLIM hubs is established. If the NAT type is symmetric, a connection to the relay server is established.

### 3.4. Concept of Monitoring Mobile App

**Figure 11:**
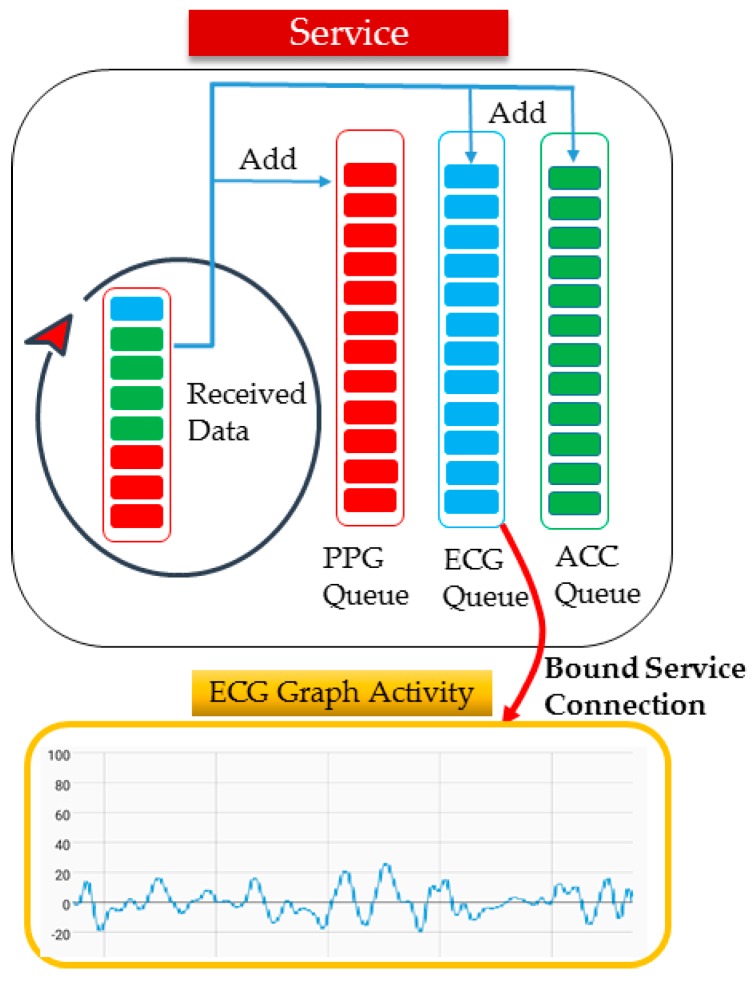
Software structure of mobile app.

Figure 11 shows the software structure of the mobile app. Because the measuring device measures various bio-signals at different sampling periods, different numbers of biometric data are contained in a single packet. For example, three PPG data, one ECG data and four ACC data are sent in a single packet. The mobile app extracts the bio-signals (PPG, ECG, ACC, etc.) contained in a single packet and stores them in a queue. Then, a graph is drawn considering the sampling period of the bio-signal. For instance, assuming that the sampling period of the PPG is three-times shorter than that of an ECG, the PPG graph is drawn three times when the ECG graph applies a single coordinate.

## 4. Detail Design of Streaming Service

### 4.1. Streaming Service between Mobile App and SLIM Hub

**Figure 12:**
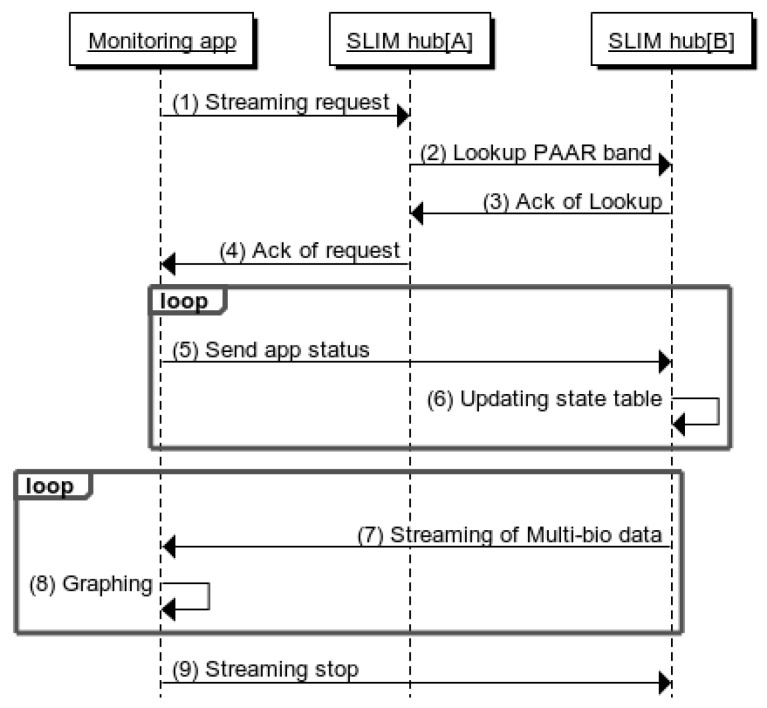
Sequence diagram of streaming service between mobile app and SLIM hub.

Figure 12 shows a sequence diagram between the mobile app and a SLIM hub. SLIM hub[A] indicates the SLIM hub registered in the caregiver’s app and SLIM hub[B] indicates the SLIM hub on the care recipient’s side. First, the mobile app sends a request to SLIM hub[A] including the PAAR band ID (1). Next, SLIM hub[A] finds SLIM Hub[B], where the device is currently located, by searching the neighbor to determine if the device is near it (2). Then, SLIM hub[B] responds to SLIM hub[A] with its IP address and status information of the measuring device in response (3, 4). That is, the caregiver can know the status of the measurement terminal located at a remote location using the mobile app. In addition, the PUB (SLIM hub[B]) does not know whether the SUB (mobile app) is normally connected and the mobile app periodically sends its current status information (5, 6). The mobile app graphs the received biometric data (7, 8) and finally sends stop message (9).

### 4.2. Streaming Service between SLIM Hub in a Private Network

**Figure 13:**
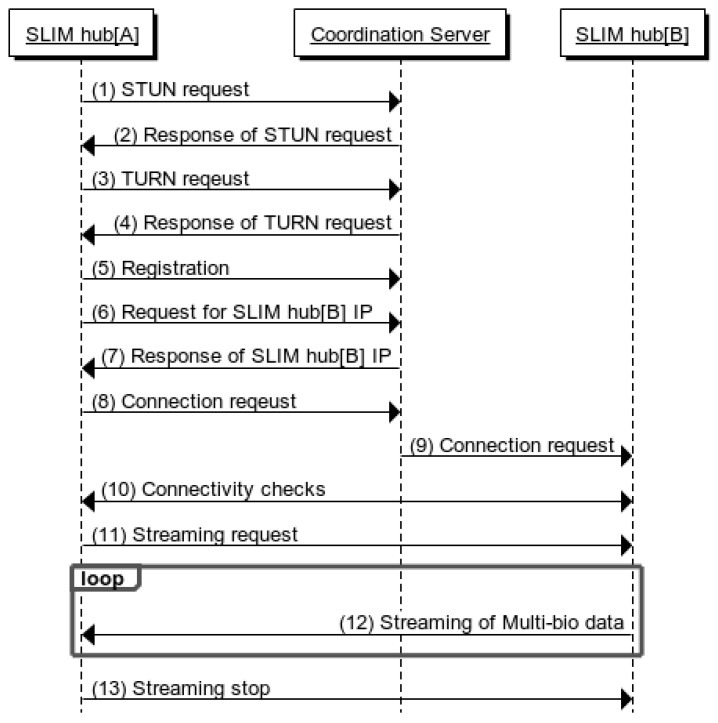
Sequence diagram of SLIM hubs in other private network.

Figure 13 shows a sequence diagram for a SLIM hub in a private network. First, the STUN protocol is used to find pairs of public IP addresses and private IP addresses mapped to the NAT device (1, 2). Then, a port allocation is requested to the TURN server using the TURN protocol (3, 4). The SLIM-Hub then sends the acquired list of addresses to the coordination server, which stores the list of SLIM hub addresses (5).

SLIM hub[A] requests the IP list of SLIM hub[B] from the server (6). The server sends the information of SLIM hub[B] to SLIM hub[A] and at the same time sends information of SLIM hub[B] to SLIM hub[A] (7). SLIM hub[A] sends a connection request message to a server (8). Then, the server that receives the request sends a connection request message to SLIM hub[B] (9). SLIM hubs[A] and [B] both send a connectivity check message to all other parties on the candidate list (10). Finally, a streaming request is sent (11) and the streaming service is started (12).

### 4.3. Streaming Service between SLIM Hub and Measurement Device

**Figure 14:**
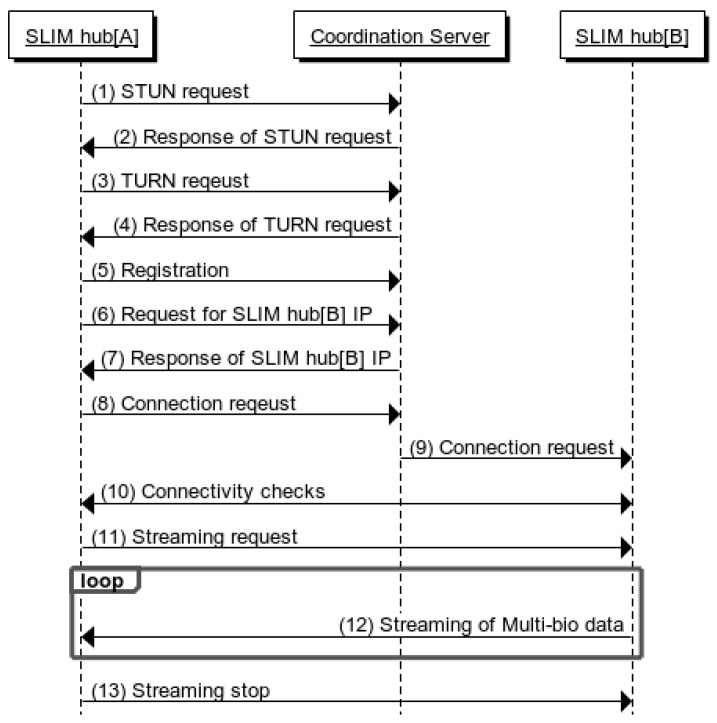
Sequence diagram of streaming service between PAAR band and SLIM hub.

Figure 14 shows a sequence diagram of the relationship between the streaming agent, module device responsible for RF communication in SLIM hub and PAAR band. First, the PAAR band periodically sends a BLE advertisement message to announce its current location (1). The RF module receives the advertisement message and stores the device information. When the streaming agent sends a request to the RF module, the RF module requests a BLE connection to the band (2, 3). Once the BLE connection is established, the measured biometric data are transmitted to the RF module through BLE communication and finally to the streaming agent (4, 5). Finally, agent sends a streaming stop message and the service ends with a BLE disconnection (6, 7).

## 5. Implementation and Performance Evaluation

### 5.1. Test Environment

**Figure 15:**
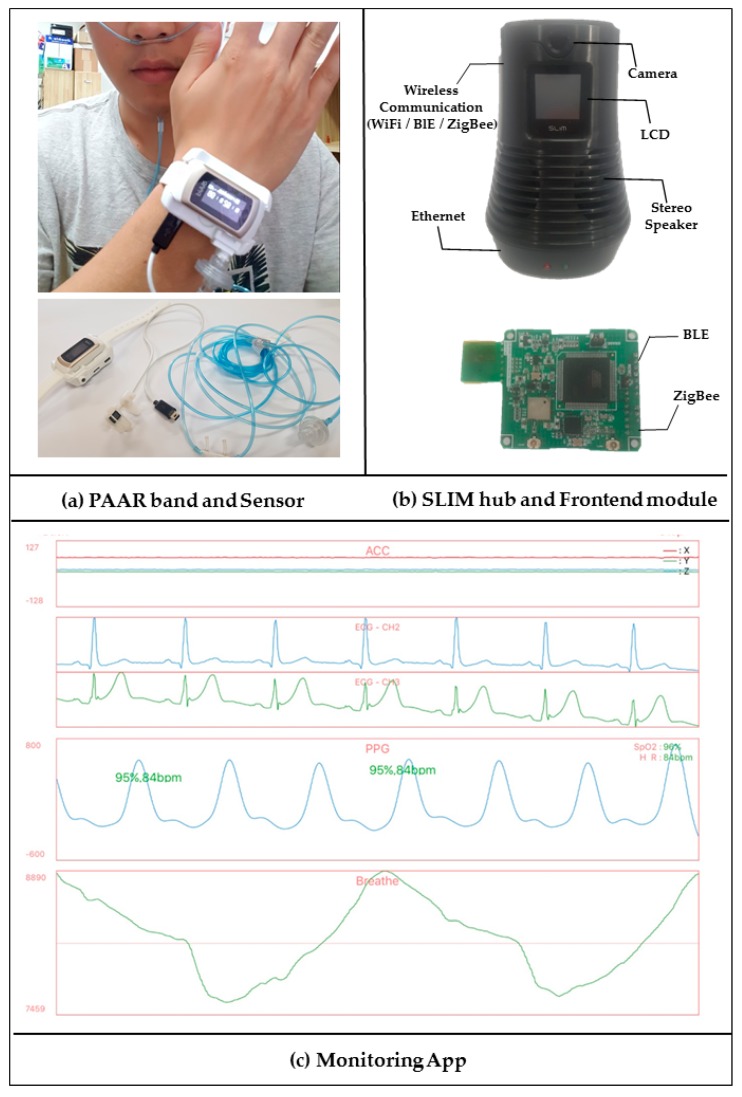
Hardware modules used in experiments. (**a**) PAAR band and Bio-signal measurement sensor; (**b**) SLIM hub and Frontend module; (**c**) Monitoring App.

Figure 15 shows the hardware module and monitoring application used in the experiment. Figure 15a shows the PAAR band, ECG, PPG and Breath measurement sensor and shows the user wearing it. Figure 15b shows the SLIM hub receiving the bio-signals measured in the band via wireless communication (there is a frontend module in the SLIM hub for RF communication). In addition, the SLIM hub provides indoor location-based services without user setting and serves as a gateway to send data acquired by the sensor to the remote caregiver using WiFi, TCP/IP, UDP. Figure 15c shows an application that enables a remote caregiver to monitor multi-bio-signals received from a SLIM hub in real time. In Section 5.2, when the app sends a streaming service request, the average time until the service is performed is measured. In Section 5.3, we evaluated the average jitter, which is the delay between packets with increasing number of receiving terminals. In Section 5.4, we evaluated connectivity between SLIM hubs in a private network environment.

### 5.2. Evaluation of Service Start Time in a Public Network

**Figure 16:**
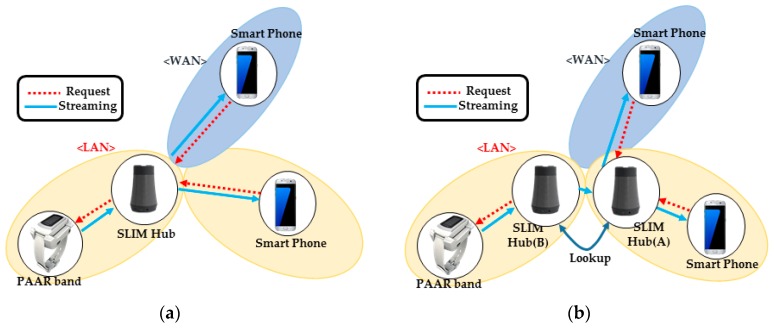
Experiment environment of service start time according to streaming request. (**a**) Without lookup neighbors; (**b**) Lookup neighbors.

Figure 16a shows that the PAAR band exists near SLIM hub registered in the app and is streamed according to the request. The registered SLIM hub can send a streaming request to the PAAR band without having to find a neighbor SLIM hub. Figure 16b shows that the PAAR band does not exist near SLIM hub registered in the app. Because of this, the registered SLIM hub finds the SLIM hub where the PAAR band is located and sends the request. In both environments, the streaming request time from the smart phone app to SLIM hub A and to SLIM hub B, was found. After that, the time until the corresponding biometric data were received was measured. This experiment shows the average time until the streaming service was made available. Both experiment environments were configured for use in LAN and WAN environments. To configure the LAN environment, a smartphone was connected to a router on the same LAN. In addition, to make the WAN environment, a smartphone was connected to a 4G network.

**Figure 17:**
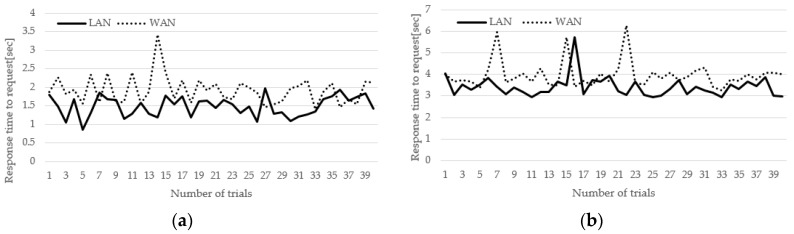
Response time to streaming request of LAN and WAN environment. (**a**) Without lookup neighbors; (**b**) Lookup neighbors.

Figure 17a shows the result of Figure 16a. The horizontal axis represents the number of trials and the vertical axis represents the service start time. The average time taken in the LAN environment was 1.49 s, with a standard deviation of 0.27 ms. In the WAN environment, it took an average of 1.92 s and a standard deviation of 0.37 ms. Figure 17b shows the result of Figure 16b. The average time taken in the LAN environment was 3.41 s, with a standard deviation of 1.49 ms. In the WAN environment, it took an average of 3.97 s and a standard deviation of 1.92 ms. This means that it takes about 2 s to find the SLIM hub where the PAAR band is located. In addition, this means that the WAN environment has a larger standard deviation than the LAN environment but the service is stable.

### 5.3. Evaluation of Jitter

**Figure 18:**
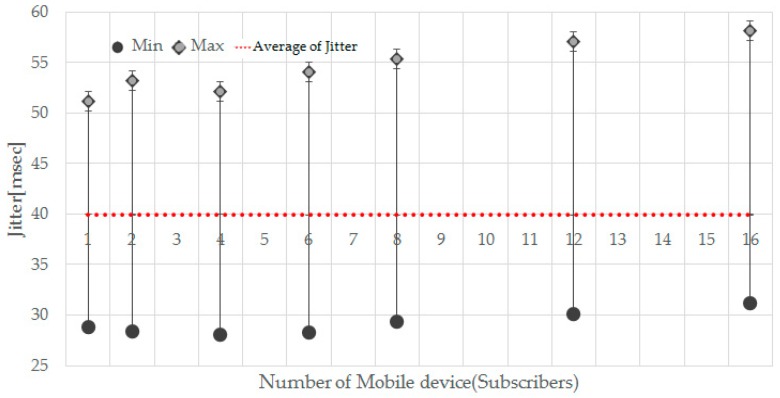
Measuring the jitter by increasing the number of mobile devices.

Jitter is the difference in packet delay. It is measuring time difference in packet inter-arrival time. That is, it is a value expressing how fast or late a signal appears compared with the reference point. The jitter was measured by receiving about 5000 biometric data on the mobile app. In addition, the jitter was measured by increasing the number of mobile device in a situation where the publisher transmits multi-bio-signals at intervals of 39.8 ms. Publishers transmit at a minimum of 27 ms, a maximum of 50 ms and an average of 39.8 ms. In Figure 18, the *x*-axis of the graph represents the number of mobile devices and the *y*-axis indicates the jitter. As the number of mobile devices increases, the min and max values tend to increase but the average jitter value is almost constant at 40 ms. Table 2 shows the measured value of the average jitter. Even if the receiving mobile device increases, there is a value in the vicinity of 40 ms. This shows that a stable streaming service is provided even if the number of subscribers increases.

### 5.4. Connectivity Check in a Private Network

**Figure 19:**
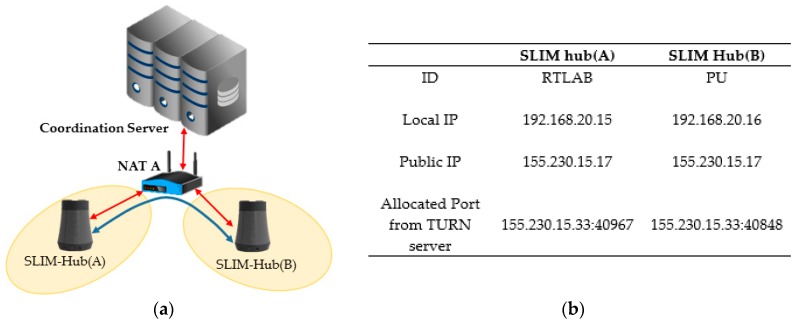
Connectivity environments of same NAT. (**a**) Configuration of experiment; (**b**) A list of SLIM hub’s ID and IP information.

To confirm the establishment of a connection between neighboring SLIM hubs in a private network, we proceeded under the same NAT environment and under a different NAT environment. Figure 19a shows that the two SLIM hubs exist under the same NAT and are in the same private network. Figure 19b shows the ID and IP list for the connection between SLIM hub.

**Figure 20:**
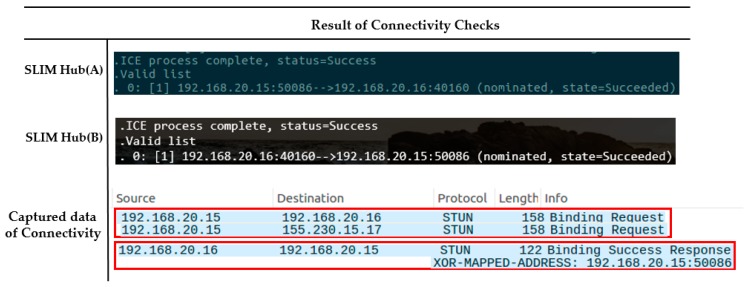
Connectivity checks in same private network.

Each SLIM hub has gathered all of its candidates, it orders them in highest to lowest priority. The SLIM hub (A) sends a request to coordination server containing the peer’s ID (PU) to obtain the IP list of the SLIM hub (B). The server sends the information of SLIM hub (B) to SLIM hub (A) and at the same time sends information of SLIM hub (B) to SLIM hub (A). Then, SLIM hubs (A) and (B) both send a connectivity check message to all other parties on the candidate list. As shown in Figure 20, the connection between the local IP of the SLIM hub in the private network was established (192.168.20.15 <-> 192.168.20.16). We used Wireshark as a packet capture tool to verify the process. The captured data in Figure 20 illustrate the connectivity check process.

**Figure 21:**
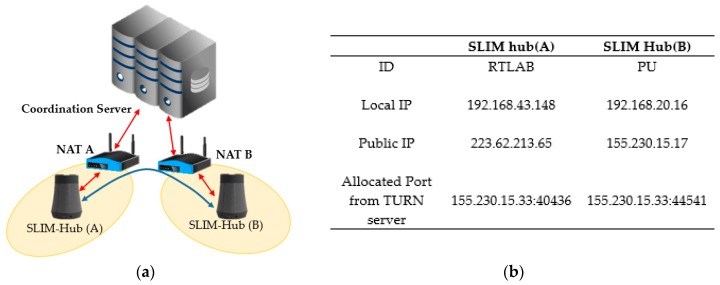
Connectivity environments of different NATs. (**a**) Configuration of experiments; (**b**) A list of SLIM hub’s ID and IP information.

**Figure 22:**
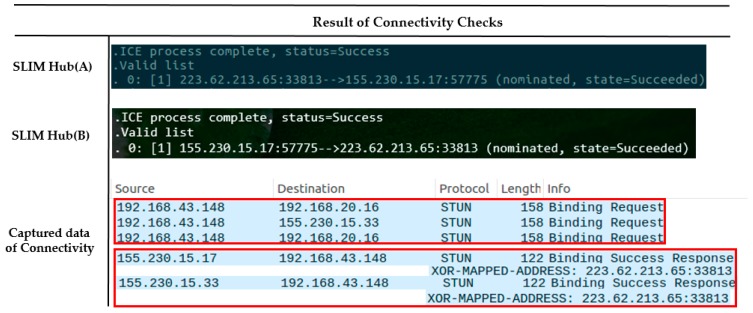
Connectivity checks in different private networks.

Figure 21a shows that the two SLIM hubs exist under a different NAT and are in a different private network. Figure 21b shows the IP list for the connection between SLIM hubs. In addition, we assume that the mapping rule of NAT equipment in the experimental environment is Endpoint-Independent Mapping (EIM). This means that if the source IP and port of the packet are the same, they can be sent to the same public IP, port regardless of the destination of the packet. That is, even if SLIM Hub A sends a packet to B’s public IP, B’s NAT device can pass packet to the mapped local IP regardless of A’s IP. As Figure 22 indicates, a connection between the public IP of the SLIM hub in the private network was established (155.230.15.17 <-> 223.62.213.65). In addition, the data captured using Wireshark for verification indicate that the connection request of each hub was sent to the candidate list of the other hub. If the mapping rule of the NAT device is not EIM, P2P communication is impossible. Therefore, the connection is established using the port assigned to the relay server.

## 6. Discussion

This study aimed at a healthcare monitoring and streaming middleware based on self-organizing middleware platform that can monitor the status of protected persons regardless of where the caregivers are located without a central server. Because the self-organizing middleware platform was developed for indoor location-based ubiquitous computing, it needed a messaging hub that can collect information of logical unit space sensors and communicate with external terminals. In the proposed middleware, the caregiver sends a streaming request to the messaging hub registered in the app, so at least one hub must be on the public network. Also, if the care recipient is near a hub that is not registered in the caregiver app, it is necessary to find the node through collaboration between the hubs. This is not a problem in the public network but in a private network environment, peer-to-peer communication between hubs is required because communication is not possible without a central server. Therefore, we propose a healthcare monitoring middleware based on self-organizing middleware platform that supports peer-to-peer communication.

The first part to discuss is related to bio-signal measurement and sensor networks. In the proposed middleware, biometric data measured by various bio signal measurement sensors are collected in PAAR band. The PAAR band is a type of smart band that allows the user to measure at any moment by connecting the measurement sensor to the wire. Although many studies use wireless communication such as Bluetooth, ZigBee and CoAP [34] protocols to measure wireless signals for building wireless sensor network (WSN), it is difficult to synchronize the biological signals from one body. For example, if the data measured by the breathing sensor during sleep and the data measured by the PPG sensor are not synchronized, it is difficult to analyze sleep apnea. Therefore, accurate synchronization is required for wireless body area network (WBAN) [35,36]. In the proposed middleware, although it is connected by wire, WBAN research using the ANT protocol is underway. The second is about transmitting the measured bio-signal to a remote location. Many studies [20,21,22] have proposed a system in which smart phones or mobile devices act as gateway devices and send data to remote locations. This has the advantage of device performance, portability and convenience but can not be monitored without a smartphone when requested at a remote location. In the proposed middleware, we tried to solve the problem by using fixed gateway equipment for each unit space. Finally, we discuss the service delivery time, connectivity and scalability of the proposed middleware in public and private networks. In Section 5.2, the average service response was evaluated by measuring the service time according to the service request in the LAN and WAN environment. In addition, we measured the time it takes to find a care recipient’s location through collaboration between hubs by placing a smart band near a messaging hub that is not registered in the caregiver app. Section 5.3 evaluated the scalability by increasing the monitoring terminal and measuring the average jitter. Since the average jitter does not change much even if the number of terminals increases by using the PUB-SUB model, biometric signals can be received at almost the same interval. In Section 5.4, we conducted peer-to-peer connectivity experiments between messaging hubs using ICE protocol in a private network environment. Depending on the type of NAT device, peer-to-peer communication may not be possible but in this case communication is performed through the relay server. Our proposed middleware will be useful for health screening by real-time monitoring of the vital signs of people who need continuous observation, such as chronic disease patients and elderly living alone.

## 7. Conclusions and Future Work

In this paper, we propose a healthcare monitoring middleware based on self-organizing middleware platform that supports peer-to-peer communication. We have tried to solve the centralized healthcare monitoring system of previous studies by self-organizing middleware platform. To evaluate the performance of the proposed middleware, we measured and analyzed the time required to start the monitoring service and the data delay according to the request. In addition, we measured time difference in packet inter-arrival time by increasing the number of mobile device. Finally, we tested the connectivity between hubs in the same private network environment. And we tested the connectivity between hubs in other private network environments. Through the proposed middleware, the caregiver can monitor the biological signal of the care recipient from the remote place. In addition, people with sleep apnea hopes to reduce the cost of visiting a sleep clinic center by sending biopsy data such as breathing, oxygen saturation and heart rate to the hospital during sleep.

As future work, we will study ANT protocol based body area networks that can synchronize multiple bio-signals to build a wireless sensor network between bio-signal measurement sensors and smart bands. And research is needed to predict [37] and distribute traffic about the publish/subscribe model used for monitoring multiple users in the public network. In addition, UDP-based publish/subscribe models should be studied for 1:N monitoring in private networks. Finally, we will conduct research related to sleep apnea that occurs during user’s sleep rather than extensive health care system and will study convenient sleep monitoring and analysis system.

## Figures and Tables

**Table 1:** A table that stores the state information of the slim hub on the coordination server.

	ID	Local IP	Public IP	Allocated Port from TURN Server
SLIM hub (A)	a1	192.168.0.1	155.230.a.b	155.230.y.z:1111
SLIM hub (B)	b1	192.168.0.2	155.230.c.d	155.230.y.z:2222

**Table 2:** Average Jitter by increasing the number of mobile devices.

Number of Mobile Device	1	2	4	6	8	12	16
Average of Jitter (ms)	39.948	39.953	39.961	39.984	39.898	39.99	39.94
